# The Interaction of REM Fragmentation and Night-Time Arousal Modulates Sleep-Dependent Emotional Memory Consolidation

**DOI:** 10.3389/fpsyg.2019.01766

**Published:** 2019-08-02

**Authors:** Gosia Lipinska, Kevin G. F. Thomas

**Affiliations:** UCT Sleep Sciences and Applied Cognitive Science and Experimental Neuroscience Team (ACSENT), Department of Psychology, University of Cape Town, Cape Town, South Africa

**Keywords:** emotional memory, emotional arousal, sleep, REM sleep, sleep-to-remember, sleep-to-forget hypothesis, noradrenaline

## Abstract

The sleep-to-forget, sleep-to-remember (SFSR) hypothesis states that the neurobiological environment provided by rapid-eye movement (REM)-rich sleep decouples the content of an emotional memory from its attendant emotional arousal. This decoupling allows *divergent* attenuation and enhancement effects (i.e., erosion of the memory’s emotional tone and simultaneous strengthening of its content). However, support for this proposal is mixed. An alternative account suggests there might be *convergent* attenuation and enhancement (i.e., elevated emotional arousal is positively coupled with enhanced emotional memory). We tested predictions emerging from the SFSR hypothesis using (a) individuals diagnosed with post-traumatic stress disorder (PTSD; *n* = 21), (b) trauma-exposed non-PTSD individuals (*n* = 19), and (c) healthy controls (*n* = 20). We included PTSD-diagnosed individuals because they typically experience altered REM sleep, impaired emotional memory, and heightened emotional arousal in response to threatening stimuli. Participants were assessed before and after both an 8-h period of polysomnographically monitored sleep and an 8-h period of waking activity. The assessment included exposure to negatively valenced, positively valenced, and neutral pictures before the 8-h delay, and a recognition task afterward. We measured emotional arousal by recording psychophysiological responses to the pictures, both pre- and post-delay. Results indicated no significant between-group differences in emotional memory accuracy or arousal. However, after a sleep-filled delay, pictures of all categories were recognized with equal accuracy, whereas after a wake-filled delay, negative pictures were recognized preferentially. Furthermore, the findings demonstrated that a sleep-filled delay was associated with attenuated emotional arousal to pictures of all categories, whereas a wake-filled delay was associated with a rise in emotional arousal across the day. Intriguingly, poorer recognition accuracy for valenced (but not neutral) pictures was predicted by an interaction of increased REM fragmentation and increased emotional arousal. In summary, we found some support for the SFSR hypothesis in the way it describes the REM- and arousal-based mechanisms that process emotional material. We also, however, found disconfirming evidence regarding the outcome of that process (i.e., sleep did not favor consolidation of emotional over neutral memory), and we demonstrated a *convergence* between attenuation of emotional arousal and weakening of emotional content relative to neutral content.

## Introduction

Emotional memories (e.g., the memory of an encounter with a snake in a forest) are comprised of an emotional arousal component (e.g., racing heart, sweaty palms, a subjective feeling of fear) and a content component (e.g., the cognitive representation of the reptile and the spatiotemporal context in which it was encountered). The *sleep-to-forget, sleep-to-remember (SFSR) hypothesis* proposes that a series of neurobiological processes (a) decouples the two components, and (b) attenuates the arousal component while consolidating the content component (van der Helm and Walker, 2011). Hence, a key tenet of this hypothesis is that these neurobiological processes allow the individual to recall the event completely without reactivating the original emotion. Furthermore, the hypothesis states that emotional memory processing occurs during rapid-eye movement (REM) sleep and in a neural environment devoid of noradrenergic activity (Walker and van der Helm, 2009).

The current study contributes to the scientific literature regarding the SFSR hypothesis. Primarily, our aim is to determine whether the strength of emotional memories is affected by the predicted loss of emotional tone. To do so, we investigate both emotional arousal and recognition accuracy for valenced information in comparison to neutral information. Novel elements of this study are that, (a) we compare day- and night-time levels of noradrenergic metabolites, thereby examining whether night-time noradrenergic activity does, in fact, modulate sleep-dependent consolidation of emotional memory, and (b) we test predictions derived from the hypothesis in a clinical sample [a group of individuals diagnosed with post-traumatic stress disorder (PTSD)] compared to two non-clinical samples [a trauma-exposed (TE) non-PTSD group and a healthy control (HC) group].

Emotional memories are formed when the organism encounters an emotion-provoking stimulus. During the encounter, the sympathetic branch of the autonomic nervous system (ANS) is activated, and a cocktail of neurochemicals, including noradrenaline, is released (McGaugh, 2004; Kerfoot and Williams, 2018). This neurochemical activation is a unique signature for the encoding of emotional memories; neutral memories (i.e., those with a content component, but no accompanying emotional arousal component) are not marked in the same way. A robust literature demonstrates that it is this neurochemical activation that, at least partially, prioritizes both the formation and the later recall of emotional memories over neutral memories (Hammerer et al., 2017).

The SFSR hypothesis proposes that a subsequent series of neurobiological events (i.e., events that occur during the period of sleep that follows soon after the emotional encounter) continues that prioritization. More specifically, during REM sleep there are activity increases in both limbic (e.g., hippocampus, amygdala) and paralimbic [e.g., anterior cingulate cortex (ACC)] structures (Maquet et al., 1996). Within this theoretical framework, these activation increases are not coincidental, but instead are synchronized via phase-locking of ponto-geniculo-occipital (PGO) waves and the theta oscillations that dominate REM sleep (Karashima et al., 2005). These oscillations drive coordinated reactivation of the various (by now widely distributed) components of the previously acquired emotional memory, leading to their integration and strengthening (Hutchison and Rathore, 2015).

Of importance here is that this reactivation occurs in an environment devoid of noradrenergic activity. Specifically, during REM sleep noradrenergic input from the locus coeruleus (input that, typically, is enhanced during the experience of stress or anxiety) is curtailed (Pace-Schott and Hobson, 2002). Instead, the brain is perfused with acetylcholine.

Hence, this hypothesis proposes that the net result of REM-dependent emotional memory consolidation is that while the content of the emotional event is strengthened and integrated into previous networks of knowledge, the emotional arousal component is attenuated. Stated differently, recall of emotional memories in healthy individuals is not associated with the same level of sympathetic activation experienced at encoding. An adaptive advantage of this hypothesized process of emotional memory consolidation is that it allows individuals to preferentially retain salient information without incurring the repeated costs of the autonomic arousal experienced during the original event (van der Helm and Walker, 2011; van der Helm et al., 2011).

Empirical studies provide mixed support for the SFSR hypothesis. For instance, whereas some find evidence for enhanced accuracy of memory for emotional compared to neutral information after a sleep-filled delay (Wagner et al., 2001, 2006; Hu et al., 2006; Sterpenich et al., 2007; Payne et al., 2008; Nishida et al., 2009; Prehn-Kristensen et al., 2009, 2017; Chambers and Payne, 2014), others find no such effect (Wagner et al., 2007; Atienza and Cantero, 2008; Sterpenich et al., 2009; Baran et al., 2012; Morgenthaler et al., 2014; Tempesta et al., 2015, 2017; Cellini et al., 2016; Jones et al., 2016, 2018; Bolinger et al., 2018; Harrington et al., 2018). Similarly, some studies find evidence for attenuated emotional arousal after a sleep-filled delay (Pace-Schott et al., 2011; van der Helm et al., 2011; Cunningham et al., 2014), whereas others find maintained (Baran et al., 2012; Groch et al., 2013; Wiesner et al., 2015) or even enhanced (Lara-Carrasco et al., 2009; Werner et al., 2015) tone.

Although the current study adds to that existing knowledge base by measuring both emotional memory accuracy and arousal in response to previously encoded emotional stimuli, we also seek to answer the important question of whether removing some of that memory’s emotional arousal (via sleep-dependent processes) compromises the relative strength of the emotional memory (where strength of memory is operationalized here as performance on tests assessing accuracy of recall or recognition for previously learned material). The SFSR hypothesis proposes that decoupling the content component from the emotional arousal component allows each to exhibit *divergent* attenuation and enhancement effects, and eventually results in enhanced strength of emotional memory. An alternative account, however, is that there is *convergent* attenuation and enhancement, so that elevated emotional arousal is mechanistically positively coupled with enhanced emotional memory. Hence, within our experimental design the presence of divergent effects would be signaled by a post-sleep increase in accuracy of memory for emotionally arousing stimuli, but an attenuation of emotional arousal in response to the same stimuli. In contrast, the presence of convergent effects would be signaled by the same post-sleep changes (either increases or decreases) in both accuracy of memory for, and emotional arousal in response to, the stimuli in question.

Furthermore, to test the purported neurobiological mechanisms within the SFSR hypothesis, we included direct measures of noradrenergic metabolites. Finally, because PTSD-diagnosed individuals experience REM sleep abnormalities (Lipinska et al., 2014; Baglioni et al., 2016) and elevated night-time noradrenergic activity (Mellman et al., 1995), and demonstrate a profound inability to decouple the emotional arousal component from the content component of emotional memories (Pole, 2007), we included a sample of such individuals in this study. We also included a HC group, and a group of TE individuals without a current diagnosis of PTSD. The latter group served as a control for trauma exposure. Because not all people who are exposed to trauma develop PTSD (Kessler et al., 1995; Kaminer et al., 2008), it is of interest to discern whether any observed effects on physiology, behavior, affect, cognition are characteristic of the diagnosis or are, more broadly, a consequence of trauma exposure.

We tested the following predictions, all derived directly from the SFSR hypothesis:

1 All participants, except those with PTSD, will have lower noradrenergic activity at night than during the day.
2 Compared to participants in the HC and TE groups, PTSD-diagnosed participants will experience greater REM sleep abnormalities.
3 All participants, except those with PTSD, will display attenuated levels of emotional arousal in response to valenced and arousing stimuli after a sleep-filled delay relative to what will be observed after a wake-filled delay.
4 All participants, except those with PTSD, will demonstrate a smaller difference in recognition accuracy for valenced and arousing stimuli in comparison to neutral stimuli after a sleep-filled rather than wake-filled delay.
5 After a sleep-filled delay, recognition accuracy for valenced and arousing (but not neutral) stimuli will be predicted by group membership (PTSD versus TE versus HC), noradrenergic activity, REM sleep parameters, and physiological measures of emotional arousal, with a notable interaction such that PTSD-diagnosed individuals who have increased night-time noradrenaline, REM abnormalities, and elevated emotional arousal will have enhanced memory for these stimuli in comparison to controls.

## Materials and Methods

### Study Design

This cross-sectional quasi-experimental study was part of a larger research program emerging from doctoral work produced by the first author (Lipinska, 2017) investigating relations between cognition, affect, and sleep in PTSD, TE, and HC groups (Lipinska and Thomas, 2017, 2019; Breen et al., 2019).

### Participants

We recruited 107 English-speaking women from Rape Crisis Cape Town Trust, and through advertisements placed in local newspapers. We screened their eligibility using the following criteria: (1) *age* – individuals below the age of 18 years and above the age of 40 years were excluded; (2) *psychopathology* – individuals diagnosed with any DSM-IV-TR (American Psychiatric Association [APA], 2000) Axis I disorder (except PTSD) were excluded, although those in the PTSD and TE groups who presented with other anxiety and mood disorders secondary to the trauma were permitted to participate; (3) *substance abuse* – individuals with a 1-year history of alcohol or other substance abuse were excluded; (4) *medication use* – individuals who, at the time of recruitment, were taking sedative medication to regulate their sleeping patterns, or who were prescribed psychoactive medication (including antidepressants), were excluded; (5) *time since trauma* – individuals who had experienced trauma more than 5 years or fewer than 6 months prior to screening were excluded; (6) *childhood trauma* – individuals who had experienced trauma at age <18 years were excluded; and (7) *neurological history* – individuals carrying neurological conditions (e.g., epilepsy, traumatic brain injury) were excluded. Each of these factors can influence sleep patterns and memory performance (Lupien et al., 1994; Stewart et al., 1998; McEwen, 1999; Harvey et al., 2003; Vermetten et al., 2003; De Bellis et al., 2005; Grigg-Damberger and Ralls, 2012).

We included smokers in the sample because, although smoking does influence sleep, the actual differences between smokers and non-smokers are relatively small. For example, smokers take approximately 5 min longer to fall asleep, experience approximately 14 min less sleep, and have approximately 6% less slow-wave sleep (SWS) than non-smokers (Zhang et al., 2006).

Sixty-six individuals met the inclusion criteria and were invited to participate further. Six withdrew after screening (due to work or other commitments), leaving a final sample of 60 participants. Each was assigned either the PTSD (*n* = 21), TE (*n* = 19), or HC (*n* = 20) group. Participants in the former two groups were survivors of sexual assault, whereas those in the HC group were required to have experienced no DSM-IV-TR PTSD criterion A traumatic event.

### Materials and Measures

#### Diagnostic and Screening Instruments

The *MINI International Neuropsychiatric Interview* (MINI version 5.0.0; Sheehan et al., 1998) confirmed diagnoses of PTSD, and assessed for the presence of other DSM-IV-TR Axis I psychiatric conditions (including substance abuse and dependence). The *Clinician Administered PTSD Scale* (CAPS; Blake et al., 1995) validated and characterized (in terms of severity of individual symptoms) the PTSD diagnosis provided initially by the MINI. The *Beck Depression Inventory – Second Edition* (BDI-II; Beck et al., 1996) characterized depression severity (we excluded potential participants in the HC group with a BDI score ≥14). *Wechsler Abbreviated Scale of Intelligence* (WASI; The Psychological Corporation, 1999) Performance IQ estimated general intellectual functioning.

#### Emotional Memory and Reactivity Stimuli

We presented participants with subsets of pictures from the *International Affective Picture System* (IAPS; Lang et al., 2008). Based on IAPS normative data, each picture we chose could be classed as either negatively valenced and highly arousing (*Negative Pictures*), positively valenced and highly arousing (*Positive Pictures*), or neutral and non-arousing (*Neutral Pictures*). Supplementary Table S1 presents descriptive data regarding valence and arousal ratings for those images.

We used E-prime software (Psychology Software Tools, Inc., Pittsburgh, PA) and a standard 19-inch computer monitor to present the pictures across three trials. On *Trial 1*, participants were asked to remember 90 pictures. On *Trial 2*, they were asked to remember 135 pictures (the Trial 1 set, plus 45 new pictures). For both trials, each picture presentation began with a fixation cross (2000 ms). The target picture was then presented for 6000 ms, followed by a blank screen (5000 ms). Across trials, stimuli were balanced for valence and arousal as well as picture characteristics (faces, scenes, non-living objects, human figures, and luminescence). On a subsequent forced-choice *Recognition Trial*, participants were asked to discriminate between pictures presented for the first time in Trial 1 and new pictures presented in Trial 2. On this trial, each of the 135 pictures the participant had seen before was presented individually for 2000 ms, with each preceded by a 2000 ms fixation cross. While the picture was on the screen, participants had to indicate whether or not the stimulus had been presented during Trial 1.

On each trial, pictures were presented in pseudorandom order so that no more than three of the same category were presented consecutively. During Trials 1 and 2, alertness-control stimuli (one of the numbers 0–9 appearing on the screen; 10 during Trial 1, 15 during Trial 2) were randomly intermixed. Participants were instructed to press the corresponding number on the keyboard during the blank screen period.

The design of this emotional memory task differs somewhat from the standard forms used in previous studies (e.g., Baran et al., 2012). A particularly novel feature of our task is that it separates assessment of emotional arousal [i.e., comparison of arousal in response to picture presentation at the pre-delay measurement point (Trial 1) with that at the post-delay measurement point (Trial 2)] from that of emotional memory (i.e., discrimination performance on the Recognition Trial). Such separation allows one to assess emotional arousal (the pure autonomic response to stimulus presentation) without the possible confounding effects of the cognitive load added by a recognition task.

#### Physiological Measures of Emotion Arousal

We used the Vrije Universiteit Ambulatory Monitoring System (VU-AMS; Version 5fs; de Geus et al., 1995; Willemsen et al., 1996) to measure autonomic arousal in response to IAPS pictures. The device took continuous recordings of electrocardiogram [ECG; eventually used to calculated heart rate (HR)], impedance cardiogram [ICG; eventually used to calculate pre-ejection period (PEP; the interval from left ventricular depolarization to the opening of the aortic valve) and left ventricular ejection time (LVET)], and skin conductance level (SCL; the average level over the measured period) data.

We gathered individual data for each of these four indices (HR, PEP, LVET, and SCL) for each IAPS picture as it was presented on each of the three trials. Following precedent set by similar previous studies (see, e.g., Cacioppo et al., 2000), measurement of each index began 3 s before stimulus onset, continued through the 6 s of stimulus presentation, and ended 2 s after stimulus offset.

Typically, HR increases for negatively valenced emotions and decreases for positively valenced emotions, with some exceptions such as happiness (Kreibig, 2010). Of note here is that the length of our period of measurement meant we were not measuring heart rate deceleration (HRD). This phenomenon occurs in the first 3 s of stimulus presentation and is measured by subtracting the minimum beats per minute (BPM) in those 3 s from a baseline BPM, taken 0.5–2 s prior to stimulus onset (Abercrombie et al., 2008). We measured an additional 5 s after this expected 3-s deceleration and used the average HR for the total 8-s period as our outcome.

Pre-ejection period is a measure of sympathetic activity that occurs in response to stress and that is not influenced by the parasympathetic branch of the ANS (Willemsen et al., 1996; Kreibig, 2010). The shorter the PEP value, the higher the degree of sympathetic autonomic arousal (Lozano et al., 2007). LVET is also a measure of the degree of sympathetic autonomic arousal: the lower the LVET value, the higher the degree of autonomic arousal. SCL provides an index of emotional arousal independent of valence (Frazier et al., 2004).

Electrocardiogram was recorded from three disposable, pre-gelled Ag-AgCl electrodes attached in a triangular, equidistant configuration on the precardium, with signals sampled at 500 Hz. We measured ICG by placing two electrodes on the back and two measuring electrodes on the chest. The ICG signal was delivered through a low-pass filter with a cut-off frequency of 60 Hz. SCL was recorded using the constant voltage method (0.5 V), sampled at 10 Hz. Ag-AgCl non-polarizable finger electrodes (6 mm diameter contact area) filled with isotonic saline gel were attached to the distal phalanx surfaces of participants’ middle and index fingers on the non-dominant hand.

#### Sleep Laboratory Equipment

Polysomnographic data collection took place in a sleep laboratory equipped with two 16-channel Nihon Kohden NeuroFax EEG9000 electroencephalographs (EEGs) adapted for sleep research. To maintain integrity of records, we used a bipolar longitudinal montage, including the bipolar derivations F3-C3, C3-P3, P3-O1 and F4-C4, C4-P4, P4-O2, in combination with a referential montage using F3-A2, C3-A2, O1-A2 and F4-A1, C4-A1, O2-A1 derivations (For more detail regarding our laboratory arrangement, see Lipinska and Thomas, 2017).

#### Subjective Sleep Measures

The *Pittsburgh Sleep Quality Index* (PSQI; Buysse et al., 1989) assessed self-reported sleep quality over the month prior to laboratory testing. Where that questionnaire provided a measure of subjective sleep quality in the home, an adapted version, the Laboratory PSQI (Lipinska and Thomas, 2017), assessed subjective sleep quality for the single experimental night in the laboratory.

#### Urinary Normetadrenaline and Metadrenaline Metabolite Collection

Urine samples allowed us to measure normetadrenaline metabolites as a measure of central nervous system noradrenergic activity. Participants provided three samples. The first was collected during the Sleep condition procedures (between midnight and 8 a.m.), and the second and third during the Waking condition procedures (between 8 a.m. and 4 p.m., and then between 4 p.m. and midnight). Each of these collections took place during a spontaneous void. So, for instance, we did not wake participants to provide a urine sample during the Sleep condition procedures, but we did allow them to provide a sample if they woke spontaneously and asked to urinate.

Urine was collected in containers holding 10 ml 6M HCl preservative and stored at standard temperatures (2°–5°) before being transferred, within 24 h, to the chemistry laboratory where volumes for the three collections were measured, creatinine levels were recorded, and aliquots obtained. The samples were then frozen at −80°, and later assayed using gas chromatography (GC) and mass spectrometry (MS) following standard procedures (Burtis et al., 2012).

### Procedure

**FIGURE 1:**
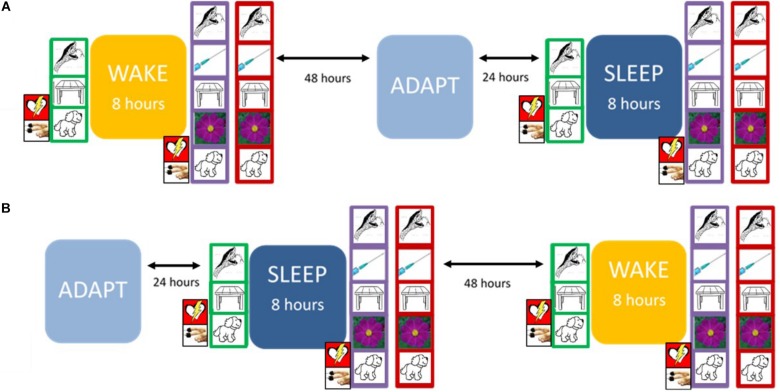
The experimental protocol. At Trial 1 (green frame), the participant encoded 90 pictures, with continuous psychophysiological recording. At Trial 2 (purple frame), the participant encoded 135 pictures (including the 90 presented previously), again with continuous psychophysiological recording. On the Recognition Trial (red frame), the participant was presented with 135 pictures and was asked to label each as either “old” (i.e., first presented during Trial 1) or “new” (i.e., first presented during Trial 2). The design was counterbalanced so that each participant experienced either **(A)** or **(B)** first. Prior to sleep on the adaptation night (ADAPT), participants completed the Pittsburgh Sleep Quality Inventory (PSQI). During the Sleep condition, they completed the Laboratory PSQI after waking.

Figure 1 presents the overall arc of the study procedure.

#### Sleep Condition

On the experimental night, participants arrived at the laboratory approximately 3 h before their regular bedtime. Immediately upon arrival, they completed informed consent procedures and were then administered some measures not relevant to the aims of this study. Approximately 20 min after entering the laboratory they were fitted with the VU-AMS and given 5 min of quiet rest to allow physiological measures to settle. They were then seated 50 cm away from a computer screen in a sound- and light-proofed room so that administration of the emotional memory task could commence.

Before beginning Trial 1, the VU-AMS recorded 2 min of quiet rest to obtain baseline readings of HR, PEP, LVET, and SCL. Participants were asked to rest the non-dominant hand, which was attached to the SCL electrodes, on the table in front of them. To ensure that ECG and ICG measures were not affected by varying respiratory behavior, they were asked to remain seated throughout the procedure and to refrain from using their phones and from making exaggerated body movements.

The Trial 1 procedure began with the participant viewing three practice pictures and one alertness-control stimulus. After the Trial concluded, the participant was disconnected from the VU-AMS. Thereafter, sleep was initiated within 30 min of their regular bedtime. Participants slept for 8 h while being monitored polysomnographically. Immediately after waking, preparing for the day, and completing another set of unrelated tasks, participants were reconnected to the VU-AMS and then administered Trial 2, and, after a 20-min break, the Recognition Trial.

During trial presentations, participants were alone in the sound- and light-proofed room, with the lights were turned off. An experimenter watched participants via infrared CCTV camera in the adjoining control room. If participants looked away from the screen, the experimenter gave a brief reminder via intercom to continue looking at the pictures.

#### Waking Condition

The formal structure of this condition was identical to that of the Sleep condition. Participants completed, in this order, Trial 1, an 8-h interval (this time, of waking activity rather than sleep), Trial 2, and the Recognition Trial. This procedure began at approximately 08h00 and concluded at approximately 18h30. They were reminded not to nap, drink caffeinated or sugary drinks, or exercise excessively during the waking interval, but were otherwise free to continue with their usual daily activities. Experimental stimuli were an alternative and formally parallel (i.e., equivalent within-category average valence and arousal) set of IAPS pictures.

### Statistical Analysis

The analyses proceeded across the following seven steps:

(1) To ensure the groups were well matched, we assessed between-group differences in sociodemographic and clinical variables using one-way ANOVAs or chi-squared tests of contingency.
(2) To ensure that circadian differences in alertness did not affect performance on the IAPS emotional memory task, we assessed between-condition differences regarding accuracy and reaction time on the alertness-control trials that were embedded within the task. Specifically, for each of the alertness-control accuracy and reaction time variables, we used a 2 × 3 mixed-design ANOVA, with Condition (Sleep, Waking) as the repeated measure and Group (PTSD, TE, HC) as the between-subjects factor, to evaluate performance.
(3) To test Hypothesis 1, we conducted a 3 (noradrenergic collection time: midnight to 8 a.m., 8 a.m. to 4 p.m., 4 p.m. to midnight) × 3 (group: PTSD, TE, HC) mixed-design ANOVA.
(4) To test Hypothesis 2, we analyzed data from three PSG outcome variables^1^ and two self-report questionnaires:
  (a) REM latency: amount of time, in minutes, before first entry into REM sleep.
  (b) REM percentage (REM%): percentage of total sleep time spent in REM sleep.
  (c) REM fragmentation: total number of arousals that occurred during REM sleep [here, we followed the American Association of Sleep Medicine (AASM; Berry et al., 2016) criteria defining REM arousals].
  (d) PSQI total score.
  (e) Laboratory PSQI total score.
  Specifically, for each of these outcome variables, we conducted a one-way ANOVA, with group as the independent variable.
(5) To test Hypothesis 3, we analyzed data from the four physiological outcome variables (HR, PEP, LVET, SCL). We employed several data cleaning techniques. To ensure the ECG recording was free from artifact, we visually inspected the inter-beat interval (IBI) time series for physiologically implausible readings. Extremely short IBIs or extremely long IBIs [following de Geus et al. (1995), we defined these as being 50% shorter or 50% longer than the mean IBI for other pictures in the same category] were marked as artifact and excluded from the analysis. On average, 1–2 such IBIs were excluded per participant. Furthermore, to calculate PEP and LVET, we manually adjusted critical points on the ECG and ICG waveforms. These points were the Q-point on the ECG waveform and the B-point on the ICG waveform. This procedure is outlined in detail elsewhere (Willemsen et al., 1996; Lozano et al., 2007). After cleaning was complete, we subtracted baseline values from each of the pre- and post-delay values for each outcome variable. Then, we took the averages of these baseline-corrected values for each picture type (Negative, Positive, Neutral). Finally, we used differences between post-delay and pre-delay baseline-corrected values as the outcome variables in four separate 2 × 3 × 3 mixed-design ANOVAs, with Condition (Sleep, Waking) and Picture Type (Negative, Positive, Neutral) as repeated measures, and Group (PTSD, TE, HC) as a between-subjects factor.
(6) To test Hypothesis 4, we used a measure of recognition accuracy based on signal detection theory (SDT; Macmillan, 2002). Within this framework, each response to a Recognition Trial stimulus may be classed as either a *hit* (i.e., a correct identification of a picture presented initially on Trial 1); a *miss* (i.e., an incorrect identification of a Trial 1 picture as having been presented initially on Trial 2); a *correct rejection* (i.e., a correct identification of a picture as having been presented initially on Trial 2, but not Trial 1); or a *false alarm* (i.e., an incorrect identification of a Trial 2 picture as having been presented initially on Trial 1). Using the sums of these four kinds of responses across the Recognition Trial stimuli, we calculated discrimination accuracy according to this sensitivity index formula:
  $$d^{\prime }=z\,\left(\text{hit}\,\text{rate}\right)-z\,\left(\mathrm{false}\,\mathrm{alarm}\,\text{rate}\right)$$
  Using that outcome variable, we then conducted a 2 × 3 × 3 mixed-design ANOVA, with Condition (Sleep, Waking) and Picture Type (Negative, Positive, Neutral) as repeated measures, and Group (PTSD, TE, HC) as a between-subjects factor.
(7) To test Hypothesis 5, we created three separate general linear models (GLMs) to test whether group membership, noradrenergic activity, REM sleep parameters, and physiological measures of emotional arousal would, after a sleep-filled delay, account for a significant proportion of the variance in recognition accuracy (*d*′) associated with Negative, Positive, and Neutral pictures, respectively. To remain within the bounds of the achieved statistical power and to control for Type I error, at the initial modeling step for each outcome variable we included a maximum of seven predictors: MAST score (an indicator of theoretically relevant substance use); BDI-II score (an indicator of theoretically relevant psychiatric status); Group (PTSD versus TE versus HC); REM fragmentation (coded as a dichotomous categorical variable, indicating whether the level of disruption was high or low); normetadrine levels at Collection Time 1 (i.e., collected between 00h00 and 08h00 as an indicator of night-time adrenergic activity), ΔHR (change in HR from pre- to post-sleep measurement; our first physiological measure of emotional arousal), and ΔLVET (change in LVET from pre- to post-sleep measurement; our second physiological measure of emotional arousal, and a measure of change in sympathetic activation across the night). We then removed non-contributing variables, starting with the most complex (e.g., 2-way interactions before main effects), and worked iteratively toward a statistically significant model that explained the most variance in the outcome.

We used SPSS (version 25.0) to complete all statistical analyses and set alpha at 0.05 for all decisions regarding statistical significance, unless noted otherwise below.

## Results

### Sample Characteristics

Analyses detected no significant between-group differences in terms of age (overall sample *M* = 25.10, *SD* = 4.45, *p* = 0.72, η^2^ = 0.01), level of education (*M* = 12.35, *SD* = 1.95, *p* = 0.11, η^2^ = 0.07), IQ (*M* = 81.62, *SD* = 13.75, *p* = 0.18, η^2^ = 0.06), income level (41 participants (68%) reported earning less than ZAR5500 per month, *p* = 0.10; *V* = 0.31), and smoking status (47 participants (78%) reported being non-smokers, *p* = 0.09; *V* = 0.28). Participants in the PTSD group reported significantly higher depression severity than those in the TE and HC groups (BDI-II *M*(*SD*) = 30.14 (6.26) versus 17.72 (7.98) versus 6.20 (3.41), *p* < 0.001, η^2^ = 0.74). More specific detail regarding sample characteristics is presented in Lipinska and Thomas (2017).

### Alertness Check

Analyses detected no significant differences, in either accuracy or reaction time, between the first session of the Sleep condition (evening session) and the first session of the Waking condition (morning session), *F*(1, 57) = 1.07, *p* = 0.35, η^2^ = 0.04, and *F*(1, 57) = 0.01, *p* = 0.92, η^2^ < 0.01, respectively, or between the second session of the Sleep condition (morning session) and the second session of the Waking condition (afternoon session), *F*(1, 56) = 0.80, *p* = 0.37, η^2^ = 0.03, and *F*(1, 56) = 3.61, *p* = 0.06, η^2^ = 0.07, respectively. Hence, we conclude there were no significant circadian differences in alertness that might have affected performance on the IAPS emotional memory task.

### Testing Hypothesis 1: Noradrenergic Activity During Sleep and Waking Conditions

Because three participants (two in the PTSD group and one in the TE group) did not follow the correct protocol for urine collection, their data were excluded from the analyses described here.

The analysis did not confirm the hypothesis, detecting no significant main effects of Collection Time or of Group, and no significant interaction effect (see Table 1). Hence, on average, (a) participants did not have significantly lower night-time than day-time noradrenergic activity, and (b) PTSD-diagnosed individuals did not have significantly higher night-time noradrenergic activity than participants in the other groups.

**TABLE 1:** Normetadrenaline collections: descriptive and inferential statistics (*N* = 57).

	**Group**						
	**PTSD**	**TE**	**HC**				
**Variable/Effect**	**(*n* = 19)**	**(*n* = 18)**	**(*n* = 20)**	***df***	***F***	***p***	**ESE**
**Repeated measure**							
Collection Time 1	17.15 (4.90)	18.74 (5.46)	16.67 (4.13)				
Collection Time 2	15.97 (3.34)	16.05 (3.37)	15.64 (3.99)				
Collection Time 3	16.17 (7.23)	17.79 (4.58)	17.26 (3.51)				
**Main/Interaction effect**							
Collection Time				2.54	2.10	0.13	0.04
Group				2.54	0.78	0.47	0.03
Collection Time × Group				4.11	0.39	0.81	0.01

*Means of square-root transformed normetadrenaline values (ug/L) are presented, with standard deviations in parentheses. TE = trauma-exposed; HC = healthy control; ESE = effect size estimate (in this case, η^2^); Collection Time 1 = midnight to 08h00; Collection Time 2 = 08h00–16h00; Collection Time 3 = 16h00–00h00.*

### Testing Hypothesis 2: Between-Group Differences in REM and Sleep Quality

Regarding objective sleep quality, analyses did not confirm the hypothesis, detecting no significant between-group differences (see Table 2). Regarding subjective sleep quality, although analyses of the PSQI data suggested that PTSD-diagnosed participants reported experiencing poorer-quality sleep over the 30 days prior to their laboratory night, analyses of the Laboratory PSQI data suggested that sleep quality was similar across groups during the laboratory night.

**TABLE 2:** Objective and subjective sleep quality: descriptive statistics and between-group comparisons (*N* = 60).

	**Group**					
	**PTSD**	**TE**	**HC**			
**Variable**	**(*n* = 21)**	**(*n* = 19)**	**(*n* = 20)**	***F*/*t***	***p***	**ESE**
**PSG outcomes**						
REM%	17.91 (5.97)	19.63 (4.22)	19.31 (4.30)	0.71	0.50	0.02
REM latency	90.10 (31.13)	97.37 (58.56)	104.60 (42.61)	0.51	0.60	0.02
REM Fragmentation	20.20 (8.56)	20.68 (11.07)	21.35 (9.60)	0.07	0.93	0.002
**Self-reports**						
PSQI total score^a^	9.65 (4.18)	5.88 (3.00)	4.16 (2.65)	13.54	<0.001^∗∗∗^	0.34
Contrast 1^b^				4.92	<0.001^∗∗∗^	1.38
Contrast 2^c^				1.53	0.13	0.61
Laboratory PSQI	5.29 (3.07)	5.26 (3.83)	4.05 (1.96)	1.09	0.34	0.04

*Means are presented with standard deviations in parentheses. TE = trauma-exposed; HC = healthy control; ESE = effect size estimate (in this case, η^2^ for *F* statistic and Cohen’s *d* for *t* statistic); PSG = polysomnograph; REM% = REM percentage; PSQI = Pittsburgh Sleep Quality Index. Because 1 participant in the PTSD group did not achieve REM sleep, degrees of freedom were (2, 56) for all PSG outcome variables. Degrees of freedom for PSQI total score were (2, 53), and for Laboratory PSQI (2, 57). ^*a*^Due to participant error in completing questionnaires, *n* = 20 for the PTSD group, *n* = 17 for TE, and *n* = 19 for HC. ^*b*^Compares the PTSD group to the combined TE and HC groups. ^*c*^Compares the TE group to the HC group. ^∗∗∗^*p* < 0.001.*

Moreover, correlational analyses suggested that, both within each group and across the entire sample, subjective sleep quality measured in the laboratory was significantly correlated with several objective PSG measures (e.g., sleep latency, sleep efficiency, number of minutes spent awake after sleep onset, and number of awakenings during the period of sleep; see Supplementary Table S2 for details).

### Testing Hypothesis 3: Emotional Arousal in Response to IAPS Stimuli in Sleep and Waking Conditions

**FIGURE 2:**
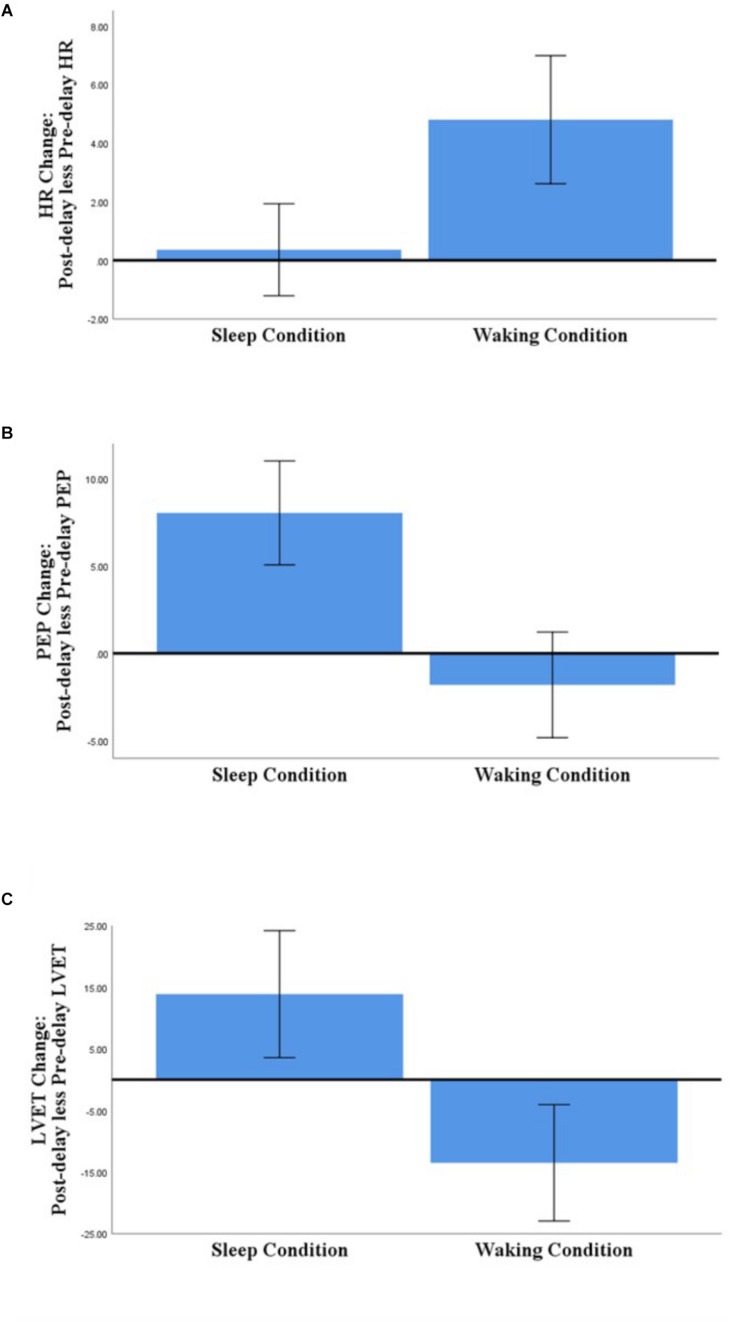
Comparison of Sleep- and Waking-associated changes in HR **(A)**, PEP **(B)**, and LVET **(C)** (post-delay less pre-delay; *N* = 58). Error bars represent 95% confidence intervals.

For each of the outcome variables, the analyses detected no significant main effects of Group and of Picture Type, and no interaction effects (see Table 3 for details of the analyses and Supplementary Tables S3, S4 for descriptive statistics). The analyses of HR, PEP, and LVET did detect a significant main effect of Condition, however (see Figure 2).

**TABLE 3:** Emotional arousal: influence of picture type and group membership across the Sleep and Waking conditions (*N* = 58).

**Variable/Effect**	**HR**			**PEP**			**LVET**			**SCL**		
	***F***	***p***	**ESE**	***F***	***p***	**ESE**	***F***	***p***	**ESE**	***F***	***p***	**ESE**
**Main effects**												
Picture Type	0.50	0.61	0.02	1.09	0.34	0.02	0.44	0.64	<0.01	0.13	0.88	<0.01
Condition	8.48	0.005^∗∗^	0.13	23.61	<0.001^∗∗∗^	0.30	11.57	0.001^∗∗^	0.17	2.13	0.15	0.04
Group	0.56	0.57	0.02	1.30	0.28	0.05	1.26	0.29	0.04	0.31	0.74	0.01
**Interaction effects**												
Picture Type × Condition	0.27	0.77	0.01	0.08	0.93	<0.01	1.10	0.34	0.02	0.44	0.65	<0.01
Picture Type × Group	1.46	0.22	0.05	1.13	0.35	0.04	1.11	0.36	0.04	0.41	0.80	0.01
Condition × Group	2.49	0.09	0.08	1.47	0.24	0.05	0.33	0.72	0.01	0.93	0.40	0.03
Picture Type × Condition × Group	1.16	0.33	0.04	0.25	0.91	<0.01	0.67	0.62	0.02	1.02	0.40	0.04

*HR = heart rate; PEP = pre-ejection period; LVET = left ventricular ejection time; SCL = skin conductance level. ESE = effect size estimate (in this case, η^2^). Degrees of freedom were: Picture Type (2, 110); Condition (1, 55); Group (2, 55); Picture Type × Condition (2, 110); Picture Type × Group (4, 110); Condition × Group (2, 55); Picture Type × Condition × Group (4, 110). ^∗∗^*p* < 0.01. ^∗∗∗^*p* < 0.001.*

Regarding those significant main effects: (a) for HR, there was no significant change across the sleep-filled delay, but there was a significant increase across the wake-filled delay; (b) for PEP, there was a substantial decrease in sympathetic activation across the sleep-filled delay alongside a small increase across the wake-filled delay; and (c) for LVET, there was a substantial decrease in sympathetic activation across the sleep-filled delay alongside an equally substantial increase across the wake-filled delay. Moreover, although the SCL results were not statistically significant, their pattern was similar to that of HR [i.e., values were maintained across the sleep-filled delay (pre-delay: *M* = −0.19, *SE* = 0.07; post-delay: *M* = 0.60, *SE* = 0.28) but increased across the wake-filled delay (pre-delay: *M* = −0.10, *SE* = 0.08; post-delay: *M* = 1.26, *SE* = 0.26)].

In summary, although these analyses did not confirm the hypothesis (i.e., there were no significant between-group or cross-stimulus differences), they did suggest that whereas sleep was associated with maintained or decreased sympathetic activation, waking was associated with an increase in such activity.

### Testing Hypothesis 4: Accuracy of Memory for IAPS Stimuli in Sleep and Waking Conditions

The analysis detected no significant effects other than a significant main effect of Picture Type (participants showed better recognition accuracy for Negative versus Positive pictures, and for Negative versus Neutral pictures) and a significant Condition × Picture Type interaction effect (see Table 4 and Supplementary Table S5 for descriptive statistics regarding hit and false alarm rates).

**TABLE 4:** Recognition accuracy: influence of picture type and group membership across the Sleep and Waking conditions (*N* = 58).

	**Group**					
	**PTSD**	**TE**	**HC**			
**Variable/Effect**	**(*n* = 19)**	**(*n* = 19)**	**(*n* = 20)**	***F***	***p***	**ESE**
**Sleep condition**						
Negative	2.09 (1.12)	2.33 (0.63)	2.47 (0.82)			
Positive	1.74 (0.96)	1.94 (0.74)	1.95 (0.77)			
Neutral	1.94 (0.91)	2.09 (0.85)	2.16 (0.88)			
**Waking condition**						
Negative	2.35 (0.90)	2.43 (1.01)	2.26 (0.79)			
Positive	1.81 (1.01)	1.82 (0.95)	1.85 (0.84)			
Neutral	1.76 (1.11)	1.74 (0.94)	1.51 (0.74)			
**Main effects**						
Picture Type				36.33	<0.001^∗∗∗^	0.40
Contrast 1^a^				47.62	<0.001^∗∗∗^	0.46
Contrast 2^b^				0.09	0.77	<0.01
Condition				1.54	0.22	0.03
Group				0.13	0.88	0.01
**Interaction effects**						
Picture Type × Condition				7.75	<0.01^∗∗^	0.12
Contrast 1^c^				11.95	<0.01^∗∗^	0.19
Contrast 2^d^				9.68	<0.01^∗∗^	0.15
Picture Type × Group				0.37	0.83	0.01
Condition × Group				1.03	0.37	0.04
Picture Type × Condition × Group				0.54	0.71	0.02

*Data presented are *d*′ scores (means with standard deviations in parentheses). TE = trauma-exposed; HC = healthy control; ESE = effect size estimate (η^2^ for omnibus tests and Cohen’s *d* for contrast analyses). Degrees of freedom: (1, 55), Condition; (2, 55), Group and Condition × Group; (2, 110), Picture Type and Picture Type × Condition; (4, 110), Picture Type × Group and Picture Type × Condition × Group. ^*a*^Negative versus Neutral pictures. ^*b*^Positive versus Neutral pictures. ^*c*^Negative versus Neutral pictures across the Sleep and Waking conditions. ^*d*^Negative versus Positive pictures across the Sleep and Waking conditions. ^∗∗^*p* < 0.01. ^∗∗∗^*p* < 0.001.*

**FIGURE 3:**
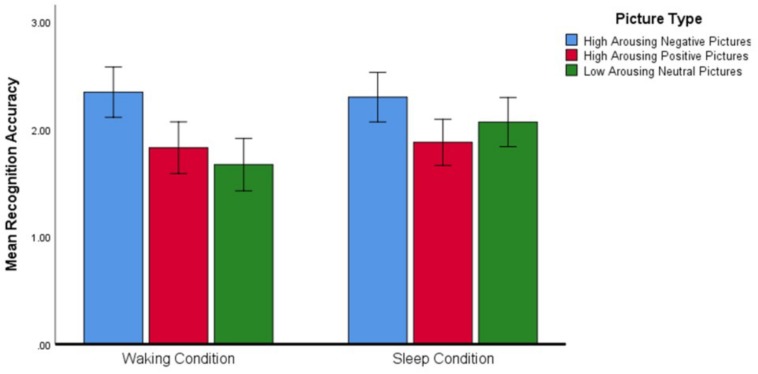
Differences in recognition accuracy (measured by the *d*′ statistic) for different types of pictures across the two experimental conditions (*N* = 58). Error bars represent 95% confidence intervals.

Regarding the significant interaction effect, follow-up analyses confirmed that the accuracy with which Negative, Positive, and Neutral pictures were recognized differed depending on the condition within which the identification was made (see Figure 3). *Post hoc* planned contrasts revealed that whereas recognition accuracy after a wake-filled delay was characterized by a significant difference between Negative–Neutral and Negative–Positive pictures (Negative pictures were remembered more accurately in both cases), there was no such difference in recognition accuracy after a sleep-filled delay. A similar series of contrasts detected no significant differences in recognition accuracy between Positive and Neutral pictures across the Sleep and Waking conditions. In summary, whereas recognition performance after a wake-filled delay was marked by a strong tendency to remember Negative pictures better than Neutral and Positive pictures, performance after a sleep-filled delay was characterized by relatively similar recognition accuracy for all three picture types.

### Testing Hypothesis 5: Modeling Negative and Neutral Memory Accuracy Using Group Status, Noradrenaline, REM Parameters, and Emotional Arousal

Table 5 presents the best-fitting model for Negative pictures’ recognition accuracy. The model, which accounted for 35.7% of the variance in the outcome, included three significant predictors: main effect of MAST score (i.e., current alcohol use), main effect of change in SCL across the night, and the interaction between REM fragmentation and change in SCL (i.e., autonomic arousal) across the night.

**TABLE 5:** General linear model: predicting recognition accuracy for Negative pictures (*N* = 58).

**Source**	**Type III SS**	***MS***	***F***	***p***	**ESE**
Corrected model	15.65	3.91	7.36	<0.001^∗∗∗^	0.36
Alcohol use	2.95	2.95	5.54	0.02^*^	0.10
REM Group	1.12	1.12	2.10	0.15	0.04
ΔSCL	2.61	2.61	4.91	0.03^*^	0.09
REM Group × ΔSCL	2.70	2.70	5.07	0.03^*^	0.09

*The categorical variable *REM Group* is based on a median split of values indicating high versus low levels of REM fragmentation. SS = sums of squares; MS = mean square.* ΔSCL = change in skin conductance level from pre- to post-sleep measurement; ESE = effect size estimate (η_*p*_^2^). ^*^p < 0.05. ^∗∗^*p* < 0.01. ^∗∗∗^*p* < 0.001.

**FIGURE 4:**
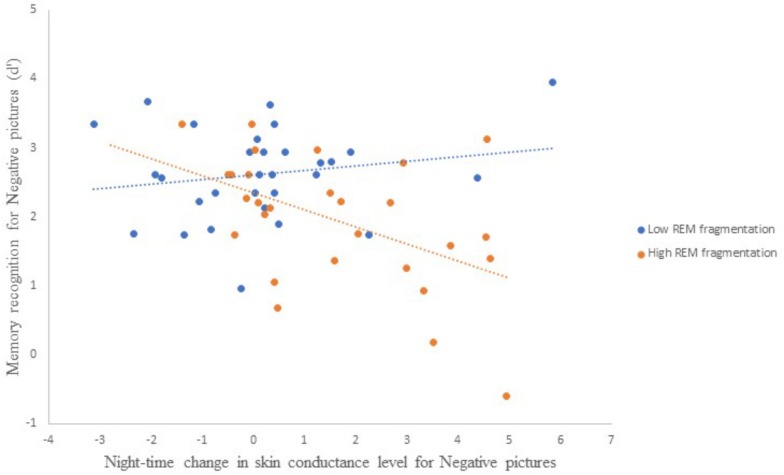
The interaction between REM fragmentation (high versus low) and night-time change in autonomic arousal (as indexed by skin conductance level) for post-sleep memory accuracy related to negative pictures.

Follow-up correlational analyses suggested that less alcohol use and more SCL change were each significantly associated with lower recognition accuracy for Negative pictures, *r* = 0.25, *p* = 0.03, and *r* = −0.31, *p* = 0.01, respectively. As to the main effect of REM fragmentation, although it was not a statistically significant predictor within the model follow-up analyses suggested that those with high levels of fragmentation had significantly lower recognition accuracy for Negative pictures than those with low levels (*M* = 1.96 ± 0.95 versus *M* = 2.62 ± 0.67), *t*(56) = 3.09, *p* = 0.003. Regarding the interaction effect, which is depicted in Figure 4, follow-up correlational analyses suggested that, within the low REM fragmentation group, there was no significant association between autonomic arousal and recognition accuracy, *r*(30) = 0.12, *p* = 0.27. In contrast, night-time increases in autonomic arousal were statistically significantly associated with poorer recognition accuracy for Negative pictures within the high REM fragmentation group, *r*(30) = −0.51, *p* = 0.003.

**FIGURE 5:**
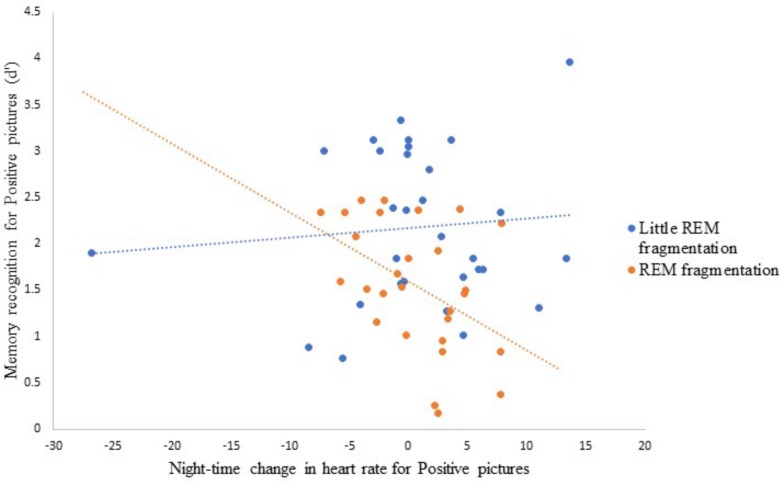
The interaction between REM fragmentation (high versus low) and night-time change in emotional arousal (as indexed by heart rate) for post-sleep memory accuracy related to positive pictures.

Table 6 presents the best-fitting model for Positive pictures’ recognition accuracy. The model, which accounted for 17.8% of the variance in the outcome, included two significant predictors: a main effect of REM fragmentation (the strongest predictor), and an interaction between REM fragmentation and change in HR (i.e., autonomic arousal) across the night. Follow-up analyses suggested that participants with high levels of REM fragmentation (*M* = 1.56, *SD* = 0.69) had significantly lower recognition accuracy for Positive pictures than those with low levels (*M* = 2.18, *SD* = 0.82), *t*(56) = 3.10, *p* = 0.003. Regarding the interaction effect, which is depicted in Figure 5, follow-up correlational analyses suggested that, within the low REM fragmentation group, there was no significant association between autonomic arousal and recognition accuracy, *r*(30) = 0.09, *p* = 0.62. In contrast, night-time increases in autonomic arousal were statistically significantly associated with poorer recognition accuracy for Positive pictures within the high REM fragmentation group, *r*(28) = −0.45, *p* = 0.02.

**TABLE 6:** General linear model: predicting recognition accuracy for Positive pictures (*N* = 58).

**Source**	**Type III SS**	***MS***	***F***	***p***	**ESE**
Corrected model	8.45	2.82	5.12	<0.01^∗∗^	0.22
REM Group	4.62	4.62	8.40	<0.01^∗∗^	0.14
ΔHR	1.52	1.52	2.77	0.10	0.05
REM Group × ΔHR	2.66	2.66	4.83	<0.03^*^	0.08

*The categorical variable *REM Group* is based on a median split of values indicating high versus low levels of REM fragmentation. SS = sums of squares; MS = mean square.* ΔHR = change in heart rate from pre- to post-sleep measurement; ESE = effect size estimate (η_*p*_^2^). ^*^p < 0.05. ^∗∗^*p* < 0.01.

Table 7 presents the best-fitting model for Neutral pictures’ recognition accuracy. The model, which accounted for 18.2% of the variance in the outcome, included one significant predictor and one trend-level predictor: a main effect of REM fragmentation (the strongest predictor) and a main effect of change in LVET (i.e., sympathetic activation) across the night. A follow-up between-group analysis suggested that participants with lower levels of REM fragmentation (*M* = 2.41, *SD* = 0.83) had significantly better recognition accuracy for Neutral pictures than those with higher levels (*M* = 1.70, *SD* = 0.76), *t*(56) = 3.33, *p* = 0.002. A follow-up correlational analysis suggested greater night-time increases in sympathetic activation were significantly associated with better recognition accuracy for Neutral pictures, *r* = −0.31, *p* = 0.01.

**TABLE 7:** General linear model: predicting recognition accuracy for Neutral pictures (*N* = 58).

**Source**	**Type III SS**	***MS***	***F***	***p***	**ESE**
Corrected model	9.08	4.54	7.34	0.001^∗∗^	0.21
REM Group	4.90	4.90	7.92	0.007^∗∗^	0.13
ΔLVET	1.96	1.96	3.16	0.08	0.05

*The categorical variable *REM Group* is based on a median split of values indicating high versus low levels of REM fragmentation. SS = sums of squares; MS = mean square.* ΔLVET = change in left ventricular ejection from pre- to post-sleep measurement; ESE = effect size estimate (η_*p*_^2^). ^*^p < 0.05. ^∗∗^*p* < 0.01.

## Discussion

The SFSR hypothesis proposes that sleep-dependent decoupling of the content component of an emotional memory from the emotional (or autonomic) arousal component eventually results in enhanced strength of the memory. In other words, if the experience of an emotional event is followed by healthy, uninterrupted sleep, memory for the content of the event will be stronger, but with weaker autonomic charge (i.e., attenuation of emotional arousal *diverges* from enhancement of memory strength). This central tenet of the hypothesis is supported by some evidence (see e.g., van der Helm et al., 2011), however some studies showing opposing evidence (see e.g., Baran et al., 2012). The current study set out to evaluate whether an alternative account of how sleep modulates emotional memory might explain why this evidence is mixed. This alternative account suggests that emotional arousal and strength of memory content *converge*, so that changes in emotional arousal are positively associated with changes in memory accuracy.

We took a stepwise approach to evaluating these differing accounts of how sleep might modulate the differing components of emotional memory. First, we examined whether emotional arousal in response to previously encoded emotional stimuli was more attenuated after a period of sleep than after a period of normal waking activity. We found that, relative to pre-delay measures, arousal either remained unchanged or decreased after a period of sleep but increased after a period of waking. These findings were not specific to any particular stimulus type (Negative, Positive, or Neutral), however.

Second, we examined whether there was a smaller difference in recognition accuracy for valenced and arousing stimuli in comparison to neutral stimuli after a sleep-filled compared to a wake-filled delay. We found that, for all participants, there was no difference in recognition memory accuracy across stimulus types after a sleep-filled delay, but that Negative pictures were recognized more accurately than Positive or Neutral pictures after a wake-filled delay.

With these results in hand, we moved on to test our main hypothesis: after a sleep-filled delay, recognition accuracy for valenced and arousing (but not neutral) stimuli will be predicted by group membership (PTSD versus TE versus HC), noradrenergic activity, REM sleep parameters, and physiological measures of emotional arousal, with a notable interaction such that PTSD-diagnosed individuals who have increased night-time noradrenaline, REM abnormalities, and elevated emotional arousal will have enhanced memory for these stimuli in comparison to controls. General linear modeling suggested that, for both Negative and Positive pictures, REM fragmentation was a key predictor of performance on the post-sleep recognition task (viz., higher levels of fragmentation were associated with lower recognition accuracy for emotionally valenced and arousing material). Most important here is that an interaction between high levels of REM fragmentation and greater *increases* in night-time sympathetic activation/autonomic arousal predicted poorer recognition accuracy for emotionally valenced and arousing material. In contrast, the model predicting performance regarding Neutral stimuli suggested more REM fragmentation and *decreases* in night-time sympathetic activation were associated with poorer recognition accuracy. Together, these findings show that, although the role of REM sleep is consistent, emotional arousal modulates memory for emotional material differently than it does for neutral material.

Another important and novel finding of this study was the difference in patterns of recognition accuracy after sleep-filled versus wake-filled delays. Whereas Negative, Positive, and Neutral pictures were recognized equally accurately after a sleep-filled delay, there was a recognition bias toward Negative pictures after a wake-filled delay. Hence, we speculate that sleep serves to attenuate arousal associated with emotional material while simultaneously elevating the arousal associated with neutral material, with the net result that all types of material are remembered equally accurately post-sleep. The adaptive value of this “resetting” of emotional memory is that the individual is not overwhelmed by valenced and highly arousing information, or by a bias toward remembering events colored by such emotional tone (a bias toward negative information and parallel increase in emotional arousal that, as we have seen, increases during the waking day). Instead, after a period of uninterrupted sleep the individual is likely to process all types of information equivalently, and can pick out salient information (i.e., information relevant to survival goals) without being prejudiced and without having cognitions distorted by the previous day’s events. Hence, maladaptive behavior patterns (e.g., hypervigilance, hyperarousal, avoidance) based on over-generalization of previous experience are less likely to occur (Pace-Schott et al., 2009).

The current findings are not consistent with a critical prediction derived from the SFSR hypothesis. That prediction states that sleep-dependent decoupling of the content component of an emotional memory from the emotional arousal component eventually results in enhanced strength of the memory (van der Helm and Walker, 2011). The theoretical framework further specifies that healthy, uninterrupted REM sleep provides a neurophysiological environment for decoupling the arousal and content components of emotional memory (i.e., there is divergent attenuation of the former and strengthening of latter). Neutral (i.e., non-valenced and non-arousing) information is not strengthened in this way. Although we found that lower levels of REM fragmentation (i.e., relatively uninterrupted REM sleep) interacted with lower levels of emotional arousal to predict accuracy of recognition for valenced stimuli, we did not find that, at the post-sleep measurement point, emotional information was remembered better than neutral information. In other words, we demonstrated a convergence between attenuation of emotional arousal and weakening of emotional content relative to neutral content.

The SFSR theoretical framework also specifies that a neural environment devoid of noradrenergic activity, such as that found during REM sleep, supports decoupling of the arousal and content components of emotional memory. However, our analyses detected no significant modulating influence of night-time noradrenergic activity on emotional memory. No previous study has provided direct evidence for the role of noradrenaline during REM sleep in emotional memory consolidation (there is only indirect evidence, from, for instance, gamma activity recorded during REM sleep; van der Helm et al., 2011). Hence, we conclude that either (a) noradrenergic activity during REM sleep is not a key neuromodulator of emotional memory consolidation, or (b) our measurement of noradrenergic metabolites was not sensitive enough to detect the predicted effects.

Our analyses also detected no effects of group membership on any of the outcomes. We recruited a group of PTSD-diagnosed individuals because literature suggests those manifesting this disorder present with persistent activation of sympathetic arousal associated with emotional memories. However, relative to TE participants and HCs, PTSD-diagnosed participants experienced few subjective or objective sleep difficulties. In fact, their sleep quality in the laboratory setting was similar to that of controls. In an earlier paper, we suggested that one possible reason for this finding is that PTSD-diagnosed participants feel safer and more comfortable in the laboratory than in their home environments (see Lipinska and Thomas, 2017, for further detail). Consistent with this lack of between-group difference in sleep quality, it is unsurprising that subsequent analyses detected no significant between-group differences in sleep-dependent processing of emotional arousal and emotional memory accuracy.

### Limitations and Directions for Future Research

Inferences and conclusions drawn from the current results must be tempered by acknowledgment of the following methodological limitations. First, although we took night-time samples of noradrenergic metabolites, we did not do so during REM sleep specifically. In fact, one might argue that a measure averaging samples taken at any time between midnight and 08h00 is not an accurate representation of noradrenergic activity during sleep. A more accurate representation would emerge from urine catheters (or blood draws, or CSF taps) taken hourly through the night. However, we attempted to make our design as non-invasive as possible, particularly given that two of our groups were comprised of women with a history of sexual assault. Other studies recruiting similar samples have used similar procedures (see, e.g., Mellman et al., 1995). Hence, our findings regarding the lack of association between noradrenergic activity during REM sleep and emotional memory consolidation must be regarded as preliminary.

Second, although we did include a measure to control for cognitive arousal, we did not control for possible circadian variation in physiological arousal. So, for example, although we speculate (based on the observed data) that sleep serves to attenuate arousal associated with emotional material while simultaneously elevating the arousal associated with neutral material, an alternative interpretation is that circadian changes in physiological arousal may affect neutral memories more than emotional memories (or vice-versa).

Third, we tested emotional memory in a somewhat unconventional manner. Unlike classic emotional memory tasks in the sleep literature, our recognition trial was separated from the initial presentation of emotional stimuli by a second presentation consisting of both “old” and “new” stimuli. This design adds additional elements (e.g., reactivation, interference) to what is present in more standard tasks. However, we argue that the design of our task was well suited to our aims in that it allowed us to assess emotional arousal (the pure autonomic response to stimulus presentation) without the possible confounding effects of the cognitive load added by a memory task (Koban et al., 2017; Koush et al., 2017). Moreover, a subsequent recognition trial, even if it follows what some might regard as an interference trial, still allows assessment of memory processed during sleep. For example, several authors have found that sleep-dependent memory processes are highlighted when an interference task is used (that is, that there is a larger sleep benefit when an interference task is inserted before retrieval after a sleep delay in contrast with a wake delay; Ellenbogen et al., 2006, 2009; Sonni and Spencer, 2015). Finally, the fact that this was an intentional rather than an incidental encoding task (i.e., the participants might have expected they were going to be tested on the learned material, and might therefore have used a different strategy than if they had no such expectation) should make no difference to the outcome: A recent meta-analysis (Lipinska et al., 2019) found that type of encoding task was not a significant moderator of the difference in memory for emotional versus neutral material after a sleep-filled delay. Overall, then, the current task is well suited to testing predictions regarding sleep-dependent memory.

Fourth, we used a quasi-experimental design, involving groups of PTSD-diagnosed individuals, TE controls, and HCs, to investigate associations between, on the one hand, emotional memory consolidation and, on the other, variability in REM sleep parameters, in noradrenaline levels, and in emotional arousal. Future studies should use experimental designs that directly manipulate REM sleep (e.g., via deprivation paradigms) and/or noradrenaline (e.g., via administered medication).

One particularly relevant direction for future research would be to replicate this study and to add a long-delay recall component (e.g., follow-up recognition testing and physiological measurement at 1 month after the initial exposure to the stimuli). This time-scale element is important because the SFSR hypothesis predicts that changes in emotional memory occur over successive nights of reactivation and reprocessing of the emotional memory, allowing for repeated decoupling of the memory’s content and emotional tone.

## Summary and Conclusion

Central to the SFSR hypothesis is the notion that healthy sleep allows preferential consolidation of memory for emotional events over that for neutral events. Contrary to that prediction, we found that, after a sleep-filled delay, previously encoded emotional and neutral stimuli were recognized with equal accuracy. Together with the finding of a recognition bias toward Negative pictures after a wake-filled delay but no such bias after a sleep-filled delay, these results suggest that one adaptive feature of sleep is to ensure that, upon waking, individuals can pick out survival-salient information without having cognitions distorted by the previous day’s events.

Our second major finding was that although uninterrupted REM sleep was a consistent predictor of better post-sleep recognition accuracy for neutral and emotional information, emotional arousal modulates memory for emotional material differently than it does for neutral material (i.e., whereas decreased night-time arousal interacts with unbroken REM sleep to produce better post-sleep emotional memory accuracy, increased night-time arousal is an independent predictor of better post-sleep neutral memory accuracy). The novelty of this finding is that it demonstrates a way in which emotional and neutral material are processed differently, with differential impact of night-time arousal on each.

In summary, we found some support for the SFSR hypothesis in the way it describes the REM- and arousal-based mechanisms that process emotional material, but we also found disconfirming evidence regarding the outcome of that process. We conclude by urging future research to interrogate individual elements of the hypothesis with more scrutiny.

## Ethics Statement

This study was carried out in accordance with the recommendations of Declaration of Helsinki (2008; www.wma.net/en/20activities/10ethics/10helsinki/) with written informed consent from all subjects. All subjects gave written informed consent in accordance with the Declaration of Helsinki. The protocol was approved by the Human Research Ethics Committee (428/2013) and the Department of Psychology Ethics Committee at the University of Cape Town.

## Author Contributions

GL designed the study, collected and analyzed the data, and wrote the first draft of the manuscript. KT involved in the study design and manuscript preparation.

## Conflict of Interest Statement

The authors declare that the research was conducted in the absence of any commercial or financial relationships that could be construed as a potential conflict of interest.

## Footnotes

1 Analyses of the complete set of PSG outcome variables are reported in Lipinska and Thomas (2017).
