# Supplementary material for: The Interaction of REM Fragmentation and Night-Time Arousal Modulates Sleep-Dependent Emotional Memory Consolidation
Source: Front Psychol. 2019 Aug 2;10:1766. doi: 10.3389/fpsyg.2019.01766 (PMC6688536; doi:10.3389/fpsyg.2019.01766)
Supplement: Supplementary file 1 [file Table_1.DOCX]

**SUPPLEMENTARY MATERIAL**

Table S1.

*Images Used in the Current Study: Self-Assessment Manikin (SAM) ratings*

|  | Picture Type | | |
| --- | --- | --- | --- |
| Condition / Trial | Negative | Positive | Neutral |
| Sleep |  |  |  |
| Trial 1^a^ |  |  |  |
| Valence | 2.25 (0.68) | 7.65 (0.48) | 5.14 (0.50) |
| Arousal | 5.76 (0.68) | 5.85 (0.66) | 3.60 (0.42) |
| Trial 2^b^ |  |  |  |
| Valence | 2.28 (0.69) | 7.63 (0.45) | 5.17 (0.48) |
| Arousal | 5.82 (0.69) | 5.17 (0.48) | 3.63 (0.46) |
| Waking |  |  |  |
| Trial 1^a^ |  |  |  |
| Valence | 2.25 (0.71) | 7.65 (0.37) | 5.11 (0.51) |
| Arousal | 5.80 (0.88) | 5.84 (0.65) | 3.65 (0.61) |
| Trial 2^b^ |  |  |  |
| Valence | 2.28 (0.69) | 7.60 (0.39) | 5.09 (0.48) |
| Arousal | 5.83 (0.92) | 5.72 (0.62) | 3.57 (0.58) |

*Note.* Data presented are *M*(*SD*). Higher SAM ratings for valence indicate an image is perceived as being more pleasant (i.e., positively valenced), whereas lower ratings indicate it is perceived as being more unpleasant (i.e., negatively valenced). The higher the SAM rating for arousal, the more arousing the image is perceived to be.

^a^The participant was presented with 30 Negative, 30 Positive, and 30 Neutral pictures.

^b^The participant was presented with 45 Negative, 45 Positive, and 45 Neutral pictures.

Table S2.

*Correlation Matrix: Associations (Spearman’s rho) between subjective and objective sleep*

*measures (N = 60)*

|  | Entire sample | PTSD | TE | HC |
| --- | --- | --- | --- | --- |
|  | (*N* = 60) | (*n* = 19) | (*n* = 18) | (*n* = 20) |
| Objective Sleep Variable | Laboratory PSQI | | | |
| Sleep Latency | .22(.08) | .22(.34) | .12(.62) | .54(.01*) |
| Sleep Efficiency | -.43(<.01**) | -.53 (.01*) | -.44(.06) | -.53(.02*) |
| WASO | .35 (<.01**) | .49(.03*) | .39(.10) | .03(.90) |
| Awakenings | .25(.06) | .45(.04*) | .19(.45) | .17(.48) |

*Note.* Data presented are rho(*p*). Values presented in boldface font represent statistically significant associations. TE = Trauma-Exposed; HC = Healthy Control; PSQI = Pittsburgh Sleep Quality Index; WASO = number of minutes spent awake after sleep onset.

**p* < .05. ***p* < .01.

Table S3.

*Emotional Reactivity to Pictures Presented on Trial 1 and Trial 2: Sleep condition (N = 60)*

|  | Trial 1 | | | Trial 2 | | |
| --- | --- | --- | --- | --- | --- | --- |
|  | PTSD | TE | HC | PTSD | TE | HC |
| Variable / Picture Type | (*n* = 21) | (*n* = 18) | (*n* = 20) | (*n* = 21) | (*n* = 18) | (*n* = 20) |
| ∆HR |  |  |  |  |  |  |
| Negative | 1.70 (3.83) | 0.16 (2.50) | 0.91 (4.54) | -0.22 (9.17) | 3.54 (5.84) | 1.33 (5.33) |
| Positive | 1.90 (3.87) | 0.88 (2.77) | 1.59 (4.38) | 0.26 (9.00) | 4.42 (6.06) | 1.66 (5.04) |
| Neutral | 2.28 (3.69) | 0.94 (2.66) | 1.94 (4.64) | 0.29 (8.88) | 4.47 (6.02) | 2.13 (7.02) |
| ∆PEP |  |  |  |  |  |  |
| Negative | 1.19 (5.67) | 0.10 (6.64)^a^ | 2.12 (13.10) | 5.03 (15.93) | 7.30 (11.81) | 14.61 (17.50) |
| Positive | 1.69 (5.49) | 0.31 (4.74)^a^ | 1.87 (14.72) | 5.59 (15.64) | 8.10 (12.46) | 14.23 (18.37) |
| Neutral | 1.71 (6.23) | -0.18 (4.78)^a^ | 2.03 (13.86) | 6.05 (15.87) | 7.44 (12.58) | 14.34 (18.67) |
| ∆LVET |  |  |  |  |  |  |
| Negative | 5.65 (44.75) | 11.98 (55.40)^a^ | -12.00 (50.27) | 28.57 (51.02) | 19.29 (65.07) | -4.51 (59.15) |
| Positive | 4.44 (42.32) | 8.01 (52.55)^a^ | -12.42 (49.08) | 29.65 (51.41) | 19.44 (64.43) | -0.40 (62.24) |
| Neutral | 3.83 (41.98) | 6.88 (54.24)^a^ | -10.79 (46.55) | 28.72 (50.67) | 19.80 (67.72) | -6.20 (63.31) |
| ∆SCL |  |  |  |  |  |  |
| Negative | -0.25 (0.53) | -0.17 (0.68) | -0.15 (0.46) | 0.38 (2.46) | 0.79 (2.17) | 0.64 (1.91) |
| Positive | -0.25 (0.52) | -0.18 (0.61) | -0.15 (0.46) | 0.34 (2.42) | 0.80 (2.14) | 0.64 (1.89) |
| Neutral | -0.26 (0.54) | -0.18 (0.61) | -0.15 (0.47) | 0.32 (2.44) | 0.83 (2.14) | 0.63 (1.90) |

*Note.* Means are presented with standard deviations in parentheses. TE = Trauma-Exposed; HC = Healthy Control; ∆HR = change in heart rate from baseline; ∆PEP = change in pre-ejection period from baseline; ∆LVET = change in left ventricular ejection time from baseline; ∆SCL = change in skin conductance level from baseline*.*

^a^n = 16.

Table S4.

*Emotional Reactivity to Pictures Presented on Trial 1 and Trial 2: Waking condition (N = 60)*

|  | Trial 1 | | | Trial 2 | | |
| --- | --- | --- | --- | --- | --- | --- |
|  | PTSD | TE | HC | PTSD | TE | HC |
| Variable / Picture Type | (*n* = 21) | (*n* = 18) | (*n* = 20) | (*n* = 21) | (*n* = 18) | (*n* = 20) |
| ∆HR |  |  |  |  |  |  |
| Negative | 1.56 (3.38) | 0.96 (2.78) | 0.06 (4.03) | 7.97 (8.47) | 4.89 (5.76) | 4.33 (11.97) |
| Positive | 1.96 (3.18) | 1.72 (2.79) | 0.89 (3.66) | 8.60 (8.61) | 5.75 (5.33) | 4.93(12.52) |
| Neutral | 1.90 (3.46) | 1.85 (3.02) | 1.14 (3.45) | 8.54 (8.37) | 5.55 (5.44) | 4.93 (12.01) |
| ∆PEP |  |  |  |  |  |  |
| Negative | 1.13 (6.53) | 0.00 (3.90) | 2.52 (13.03) | -0.49 (11.66) | -2.13 (9.27) | 1.42 (21.59) |
| Positive | 0.83 (6.44) | -0.03 (4.03) | 2.41 (12.45) | -0.57 (10.95) | -1.20 (8.78) | 1.67 (21.02) |
| Neutral | 1.00 (5.92) | -0.44 (4.48) | 3.24 (12.51) | -0.50 (11.50) | -1.58 (9.18) | 0.93 (21.48) |
| ∆LVET |  |  |  |  |  |  |
| Negative | 5.70 (54.41) | 4.22 (34.53) | -5.85 (47.18) | -5.61 (60.86) | -18.47 (39.05) | -16.16 (63.57) |
| Positive | 7.99 (55.62) | 2.23 (34.79) | -5.51 (46.52) | -3.70 (64.01) | -18.65 (33.68) | -18.44 (66.83) |
| Neutral | 4.99 (52.89) | 3.90 (35.17) | -4.54 (50.98) | -5.58 (66.27) | -18.69 (33.65) | -14.80 (65.23) |
| ∆SCL |  |  |  |  |  |  |
| Negative | -0.17 (0.67) | -0.06 (0.45) | -0.06 (0.60) | 1.71 (2.37) | 0.96 (2.14) | 1.07 (1.28) |
| Positive | -0.18 (0.68) | -0.06 (0.45) | -0.55 (0.62) | 1.71 (2.32) | 1.00 (2.17) | 1.06 (1.28) |
| Neutral | -0.18 (0.68) | -0.08 (0.45) | -0.06 (0.61) | 1.73 (2.33) | 1.01 (2.15) | 1.05 (1.27) |

*Note.* Means are presented with standard deviations in parentheses. TE = Trauma-Exposed; HC = Healthy Control; ∆HR = change in heart rate from baseline; ∆PEP = change in pre-ejection period from baseline; ∆LVET = change in left ventricular ejection time from baseline; ∆SCL = change in skin conductance level from baseline*.*

^a^n = 16.

Table S5.

*Emotional Memory (Recognition): Descriptive statistics for hits and false alarms after a sleep- and wake-filled delay (N = 60)*

|  | Sleep Condition | | | Waking Condition | | |
| --- | --- | --- | --- | --- | --- | --- |
|  | PTSD | TE | HC | PTSD | TE | HC |
| Picture Type / Outcome Variable | (*n* = 21) | (*n* = 19) | (*n* = 20) | (*n* = 21) | (*n* = 19) | (*n* = 20) |
| Negative |  |  |  |  |  |  |
| Hit rate | 25.74 (4.87) | 26.79 (2.70) | 26.60 (3.90) | 25.52 (4.63) | 26.42 (3.99) | 25.15 (3.69) |
| False alarm rate | 3.58 (3.19) | 2.95 (2.37) | 2.40 (1.90) | 2.33 (2.08) | 2.68 (2.52) | 2.15 (1.84) |
| Positive |  |  |  |  |  |  |
| Hit rate | 22.74 (5.10) | 22.26 (5.69) | 23.20 (3.96) | 23.52 (5.18) | 24.63 (4.14) | 23.00 (4.93) |
| False alarm rate | 3.16 (2.61) | 2.37 (1.95) | 2.25 (1.80) | 3.38 (3.14) | 4.05 (3.39) | 2.70 (2.03) |
| Neutral |  |  |  |  |  |  |
| Hit rate | 22.21 (4.34) | 22.21 (5.40) | 22.95 (5.43) | 22.67 (5.62) | 22.95 (4.62) | 20.05 (5.42) |
| False alarm rate | 2.16 (2.27) | 1.58 (1.22) | 1.55 (1.36) | 3.09 (2.59) | 3.26 (2.94) | 2.65 (1.69) |

*Note.* Means are presented with standard deviations in parentheses.
